# Alien suns reversing in exoplanet skies

**DOI:** 10.1038/s41598-022-11527-8

**Published:** 2022-05-19

**Authors:** Xinchen Xie, Hwan Bae, John F. Lindner

**Affiliations:** 1 Physics Department, The College of Wooster, Wooster, OH 44691 USA; 2 Physics and Astronomy, University of North Carolina at Chapel Hill, Chapel Hill, NC 27599 USA; 3 Department of Physics, North Carolina State University, Raleigh, NC 27695 USA

**Keywords:** Exoplanets, Computational astrophysics, Applied mathematics

## Abstract

Earth’s rapid spin, modest tilt, and nearly circular orbit ensure that the sun always appears to move forward, rising in the east and setting in the west. However, for some exoplanets, solar motion can reverse causing alien suns to apparently move backward. Indeed, this dramatic motion marginally occurs for Mercury in our own solar system. For exoplanetary observers, we study the scope of solar motion as a function of eccentricity, spin–orbit ratio, obliquity, and nodal longitude, and we visualize the motion in spatial and spacetime plots. For zero obliquity, reversals occur when a planet’s spin angular speed is between its maximum and minimum orbital angular speeds, and we derive exact nonlinear equations for eccentricity and spin–orbit to bound reversing and non-reversing motion. We generalize the notion of solar day to gracefully handle the most common reversals.

## Introduction

The recent surge in exoplanet discoveries expands our inventory of solar systems even as it challenges our understanding of them. Many exoplanets have unusual orbits, including those around multiple star systems^1,2^. Such bizarre solar dances imply exotic sunrises and sunsets, as when a planet orbits one star that orbits another, so that when the planet is opposite the two stars it experiences two sunsets and sunrises per day, but when it is between the two stars it experiences constant daylight.

In this article, we investigate the range of apparent solar motion for planets orbiting single stars in simple models using theory and computation. In particular, we explore the ranges of orbital eccentricity, spin–orbit ratio, and axial tilt that permit apparent solar reversals, an especially striking motion: not only can suns stand still in the sky, they can sometimes move backward. Planets in plausible pseudosynchronized rotations or spin–orbit resonances should experience solar reversals. Although the dynamics of most exoplanets are not yet fully known, we begin locating some exoplanets in the abstract parameter space of solar reversals.

We first focus on the important special case of zero tilt and derive exact nonlinear equations delimiting the range of eccentricity and spin–orbit ratio that allows solar reversals. We note that Mercury is just inside this range. Spacetime “coil” plots illustrate important examples. We next extend these results numerically to planetary tilt, parameterized by obliquity and nodal angle. Spatial “ball” plots illustrate representative cases. We then suggest a reversal-aware generalization of a solar day and consider implications of reversals for civilizations that witness them. Finally, we qualitatively discuss chaotic and multiple star systems and locate the current work in a broader context.

## Without tilt

A planet of mass *m* orbits a fixed star of mass *M* in an ellipse of radii $r_- \le r \le r_+$ and speeds $v_+ \le v \le v_-$, where subscripts ± indicate periapsis and apoapsis values. Its constant angular momentum magnitude

1 $$\begin{aligned} L = m r_\perp v = m r_\pm v_\pm \end{aligned}$$

and energy

2 $$\begin{aligned} E = \frac{1}{2} m v^2 - \frac{GMm}{r} = \frac{1}{2} m v_\pm ^2 - \frac{GMm}{r_\pm }. \end{aligned}$$

The difference in energies between the apopapsis and periapsis is

3 $$\begin{aligned} 0 =\frac{1}{2}m\left( v_+^2 - v_-^2 \right) - GMm\left( \frac{1}{r_+} - \frac{1}{r_-} \right) . \end{aligned}$$

Eliminate the speeds with the corresponding angular momenta to get

4 $$\begin{aligned} 0 =\frac{1}{2}\frac{L^2}{m^2}\left( \frac{1}{r_+^2} - \frac{1}{r_-^2} \right) - GM\left( \frac{1}{r_+} - \frac{1}{r_-} \right) , \end{aligned}$$

and solve for the specific angular momentum

5 $$\begin{aligned} \frac{L}{m} = \sqrt{\frac{2GM}{1/r_+ + 1/r_-}} = \sqrt{\frac{2GM}{r_+ + r_-}\,r_+ r_-}. \end{aligned}$$

If the larger orbital radius

6 $$\begin{aligned} a = \frac{r_+ + r_-}{2}, \end{aligned}$$

and the orbital eccentricity

7 $$\begin{aligned} e = \frac{r_+ - r_-}{2a}, \end{aligned}$$

then the extreme radii

8 $$\begin{aligned} r_\pm = a(1\pm e). \end{aligned}$$

**Figure 1:**
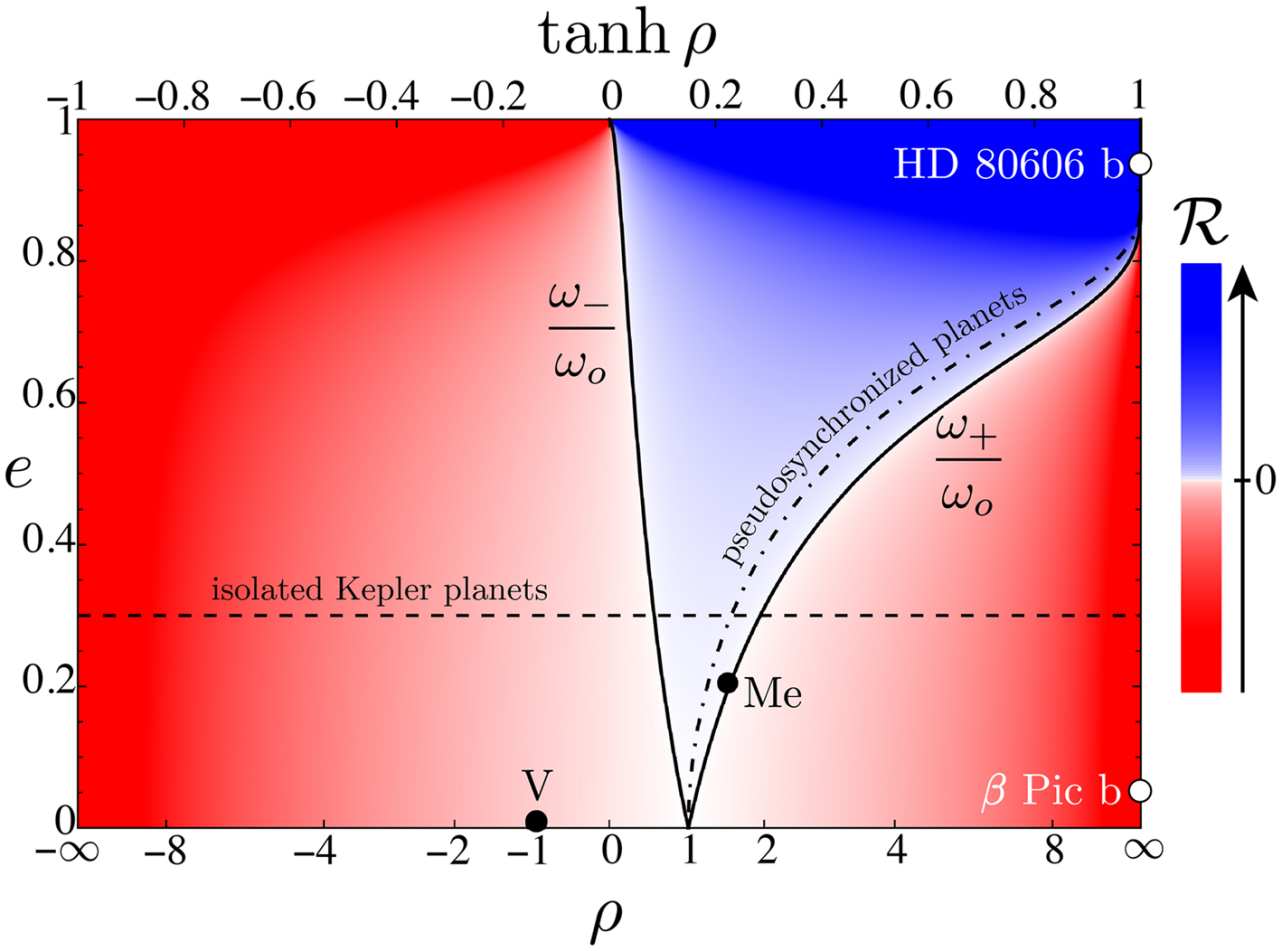
Reversals plot. Spin–orbit ratio $\rho = \omega _s/\omega _o$ increases rightward and orbital eccentricity *e* increases upward for zero tilt. Red-white-blue background colors represent the product ${\mathscr {R}}$ of the differences of the planet’s spin angular speed and its extreme orbital angular speeds at apoapsis and periapsis; red means no apparent solar reversals, blue means reversals, and the saturation indicates the reversal magnitude. Black dots are planets Venus and Mercury; white dots are exoplanets $\beta$ Pictoris b and HD 80606 b. Dashed horizontal line indicates numerous isolated Kepler planets with as yet undetermined spins. Dot-dashed curve indicates potentially pseudosynchronized planets, all of which would undergo reversals.

Hence the extreme orbital angular speeds are

9 $$\begin{aligned} \omega _\pm =\frac{v_\pm }{r_\pm } = \frac{L}{m r_\pm ^2} = \sqrt{\frac{2GM}{r_+ + r_-}\frac{r_\mp }{r_\pm ^3}} = \omega _o \sqrt{\frac{1\mp e}{(1\pm e)^3}}, \end{aligned}$$

where the *mean* orbital angular speed

10 $$\begin{aligned} \omega _o = \sqrt{\frac{GM}{a^3}}. \end{aligned}$$

Assuming constant angular spin $\omega _s$ and zero obliquity, apparent solar reversals occur when the spin angular speed is between the minimum and maximum orbital angular speeds,

11 $$\begin{aligned} \omega _{-}< \omega _s < \omega _{+} \end{aligned}$$

or

12 $$\begin{aligned} \sqrt{\frac{1 - e}{(1 + e)^3}}< \rho < \sqrt{\frac{1 + e}{(1 - e)^3}}, \end{aligned}$$

where the spin–orbit ratio $\rho = \omega _s/\omega _o$. The dimensionless function

13 $$\begin{aligned} {\mathscr {R}} = -(\omega _s - \omega _{-}) (\omega _s - \omega _{+})/\omega _o^2 = -(\rho - \rho _{-}) (\rho - \rho _{+}) = -\left( \rho - \sqrt{\frac{1 - e}{(1 + e)^3}}\right) \left( \rho - \sqrt{\frac{1 + e}{(1 - e)^3}} \right) \end{aligned}$$

quantifies the reversal, where ${\mathscr {R}} < 0$ means a sun always appears to move one-way in the sky, and ${\mathscr {R}} > 0$ means its movement sometimes appears to reverse.

**Figure 2:**
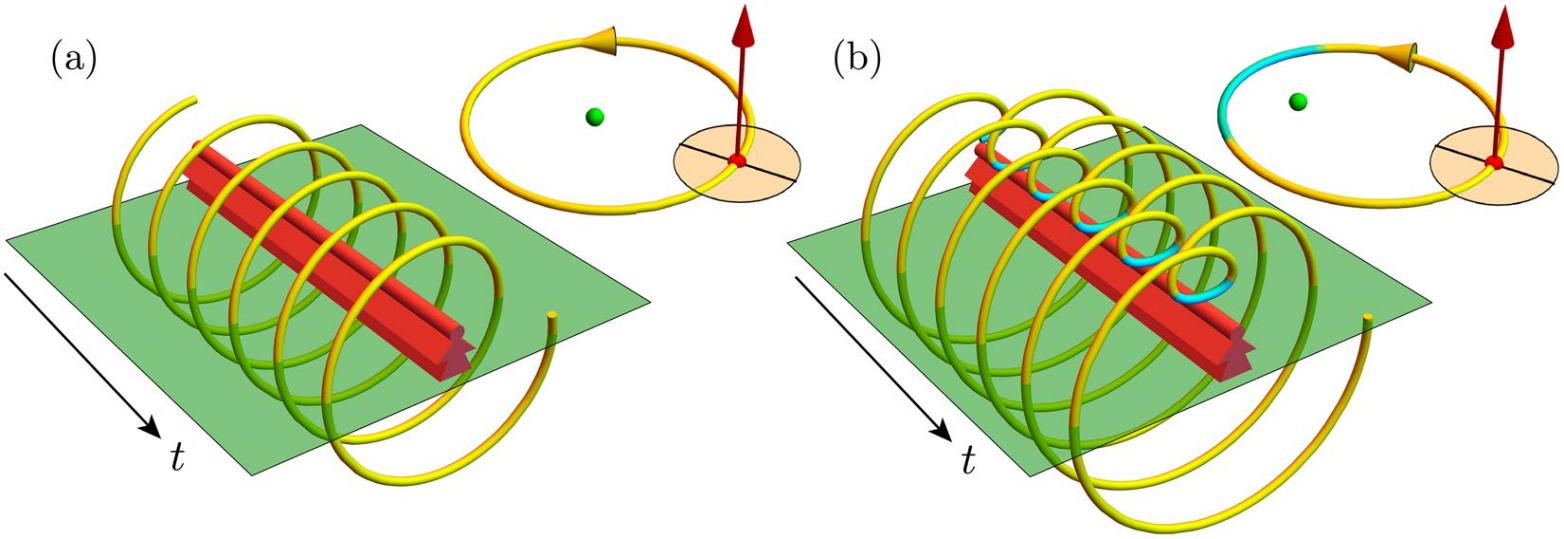
Coil plots. Spacetime extrusions (red) represent equatorial observers on their local horizons (green). (**a**) Earth-like planet with $\{\rho ,e \} = \{2, 0.1\}$, with orbit (top right) and apparent solar motion (bottom left) where sun apparently rises in the east and sets in the west with no reversals. (**b**) Exoplanet with $\{2, 0.5 \}$, with orbit (top right) and apparent solar motion (bottom left), including reversals (cyan) near periapsis and noon. Equatorial observer at the antipodes would not see the solar reversals.

**Figure 3:**
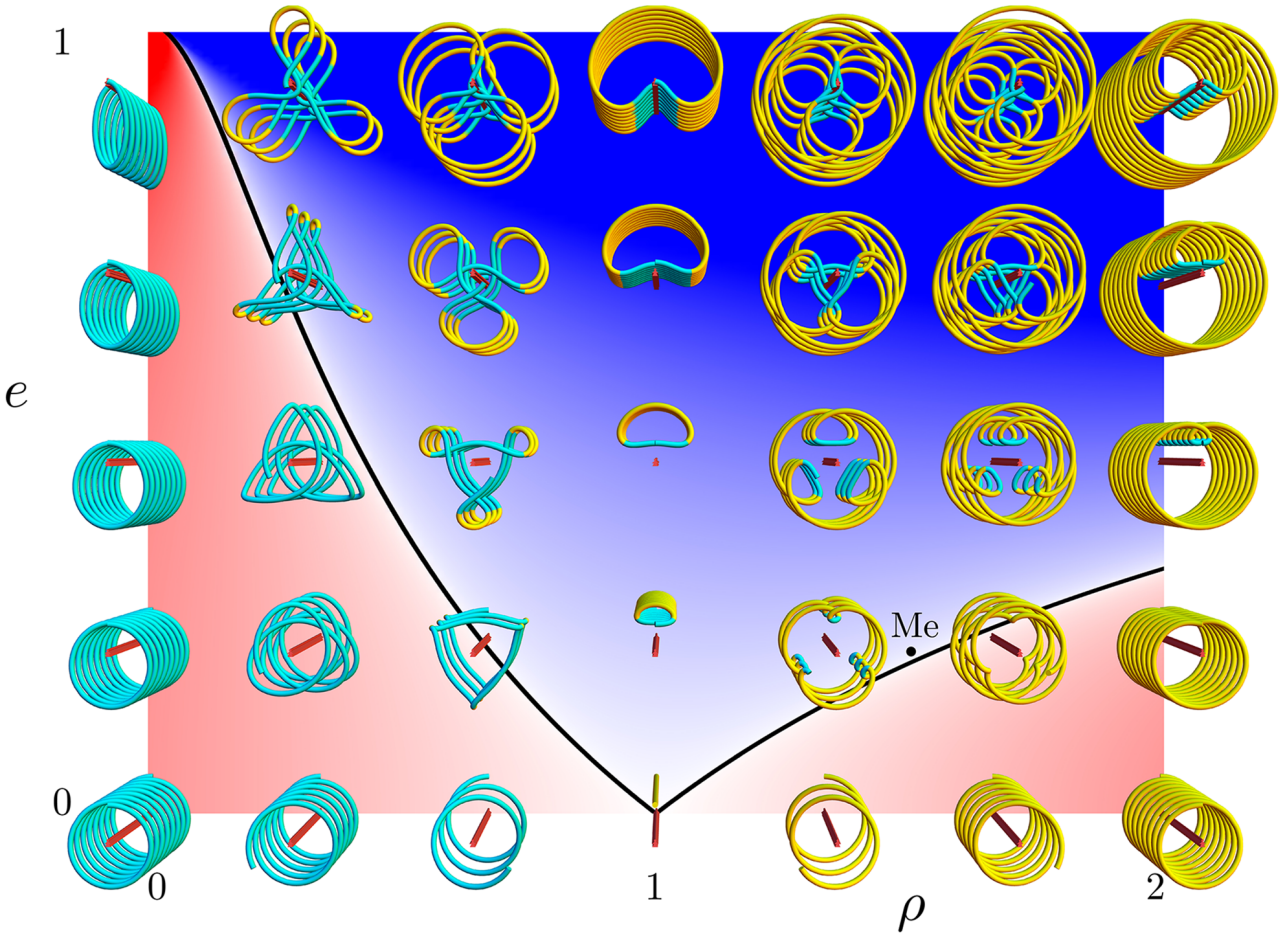
Reversals and coils. Spin–orbit ratio $\rho = \omega _s/\omega _o$ increases rightward, orbital eccentricity *e* increases upward, and time *t* increases outward. Red rods represent planetary observers, and coils represent apparent position and angle of their suns for 8 orbits. Yellow and cyan indicate apparent clockwise and counterclockwise motion, which reflect reversals in coils with both colors. The orbits of many exoplanets circularize and tidally lock at $\{\rho , e \} = \{1, 0\}$, where a sun appears fixed directly overhead. Mercury is at $\{1.5, 0.21\}$, just inside the reversal region.

**Figure 4:**
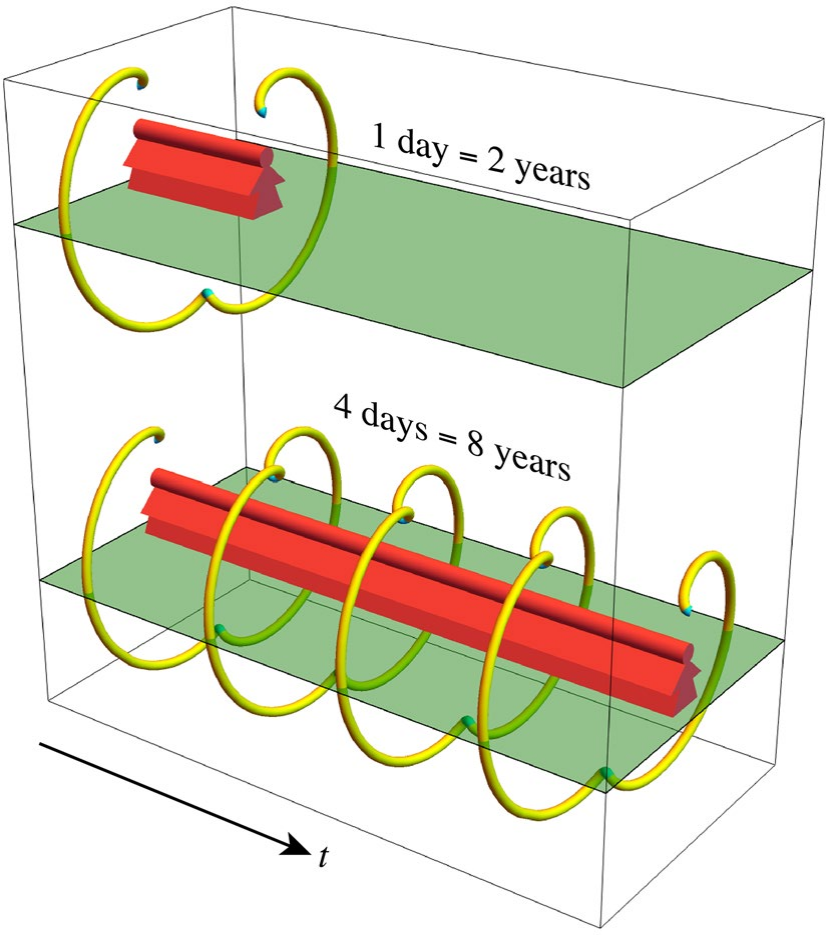
Mercury coil plot. On Mercury, one (solar) day lasts two years, and an equatorial observer (red) witnesses a small solar reversal (cyan) once a year.

Figure 1 summarizes the possibilities. Solid black curves are plots of orbital eccentricity *e* (ordinate) versus critically large and small spin–orbit ratios $\rho _\pm = \omega _\pm /\omega _o$ (abscissa). Colors code reversals ${\mathscr {R}}$, with red negative, blue positive, and saturation decreasing with magnitude. Dots indicate Venus and its unusual retrograde spin^3,4^, Mercury and its spectacular 3–2 resonance^5^, and $\beta$ Pictoris b^6^, the first exoplanet with a measured spin–orbit ratio. Mercury is just inside the blue reversal region. Other solar system planets would appear near $\beta$ Pictoris b. Horizontal dashed line indicates isolated Kepler planets with typical eccentricities $e\sim 0.3$, which experience reversals for spin–orbit ratio $\rho \sim 1$ (while Kepler multiples tend to have nearly circular orbits)^7,8^. Dot-dashed curve indicates pseudosynchronized planets^9^, such as the eccentric exoplanet HD 80606 b, all of which would experience reversals.

We visualize the apparent solar motion using Mathematica^10^ to numerically integrate the nonlinear polar equations of motion

14a $$\begin{aligned} r \ddot{\theta }+ 2 \dot{r} {\dot{\theta }} = a_\theta&= 0, \end{aligned}$$

14b $$\begin{aligned} \ddot{r} - r {\dot{\theta }}^2 = a_r&= -G M / r^2 \end{aligned}$$

starting at the apoapsis $r = r_+$, $\theta = 0$, $\dot{r} = 0$, ${\dot{\theta }} = \omega _+$ and compute the angle

15 $$\begin{aligned} \alpha = \omega _s t - \theta \end{aligned}$$

and distance *r* of the sun with respect to an equatorial observer (neglecting the planet’s radius), as in the Fig. 2 spacetime “coil” plots. A $2+1$ dimensional spacetime extrusion (red) represents the observer on the local horizon (green). A tube represents the apparent solar motion, both forward (yellow) and backward (cyan). Inset (top-right) represents orbital motion in the solar system’s reference frame, where spin (red arrow) is perpendicular to the orbits and dot (green) is the sun. Figure 2a illustrates an Earth-like planet whose sun apparently rises in the east and sets in the west with no reversals; parameters are $\{\rho , e \} = \{2, 0.1\}$. Figure 2b illustrates a non-Earth-like planet with a more eccentric orbit whose sun rises in the east and sets in the west but reverses at every noon and every perihelion (although equatorial observer at the antipodes would not see these solar reversals); parameters are $\{\rho , e \} = \{2, 0.5\}$.

We demonstrate the range of possibilities by drawing coil plots perpendicular to a grid of spin–orbit ratio and eccentricity $\{\rho , e \}$, as in Fig. 3. Red-white-blue background colors again code the reversal ${\mathscr {R}}$. Red extrusions indicate observers, with time increasing outward for 8 orbital periods. For tidally-locked planets in circular orbits, $\{\rho , e \} = \{1, 0\}$ and their suns appear to hang motionless in their skies; as eccentricity increases and the orbital speed varies, their suns appear to move backward and forward, even rising and setting in the east followed by rising and setting in the west near $\{\rho , e \} = \{1, 0.9\}$. For a given orbital eccentricity *e*, only a range of spin–orbit ratios between $\rho _\pm = \omega _\pm /\omega _o$ produce reversals (cyan and yellow).

Mercury is just inside the reversal region, and Fig. 4 focuses on its coil plot (neglecting perturbations due to asphericity, other planets, general relativity, and so on). On Mercury, one (solar) day lasts 2 years, and once a year an equatorial observer witnesses a brief solar reversal, surely a special day for any future inhabitants.

**Figure 5:**
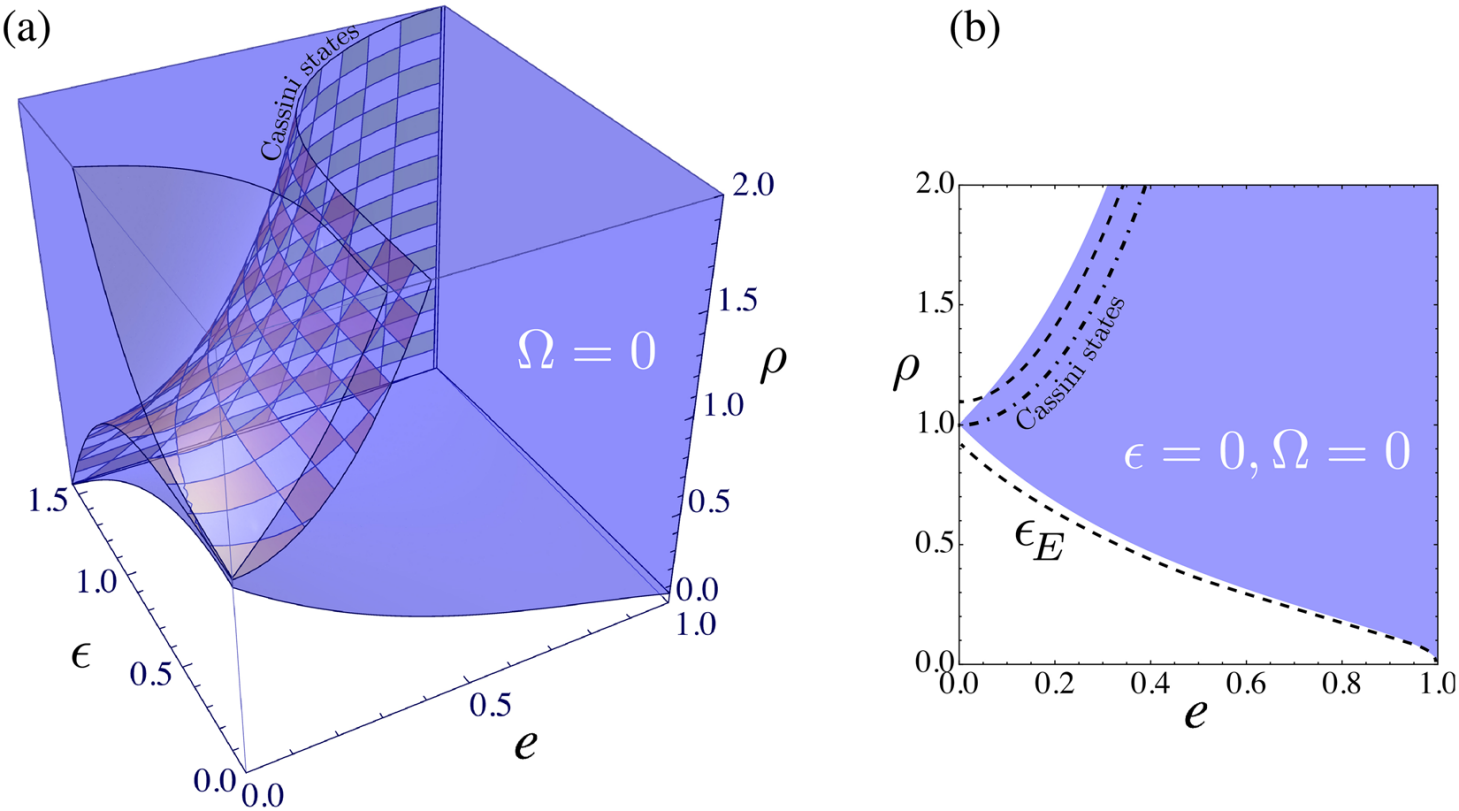
Obliquity and reversals. (**a**) 3D region where eccentricity *e*, spin–orbit ratio $\rho = \omega _s/\omega _o$, obliquity $\epsilon$, and nodal angle $\Omega = 0$ cause apparent solar reversals. Checkered surface indicates Cassini states. (**b**) 2D cross sections at $\epsilon = 0$ (solid blue) and Earth’s $\epsilon _E = 0.4$ (dashed black lines) show that small tilts only weakly affect the reversals. Dot-dashed curve indicates Cassini states.

**Figure 6:**
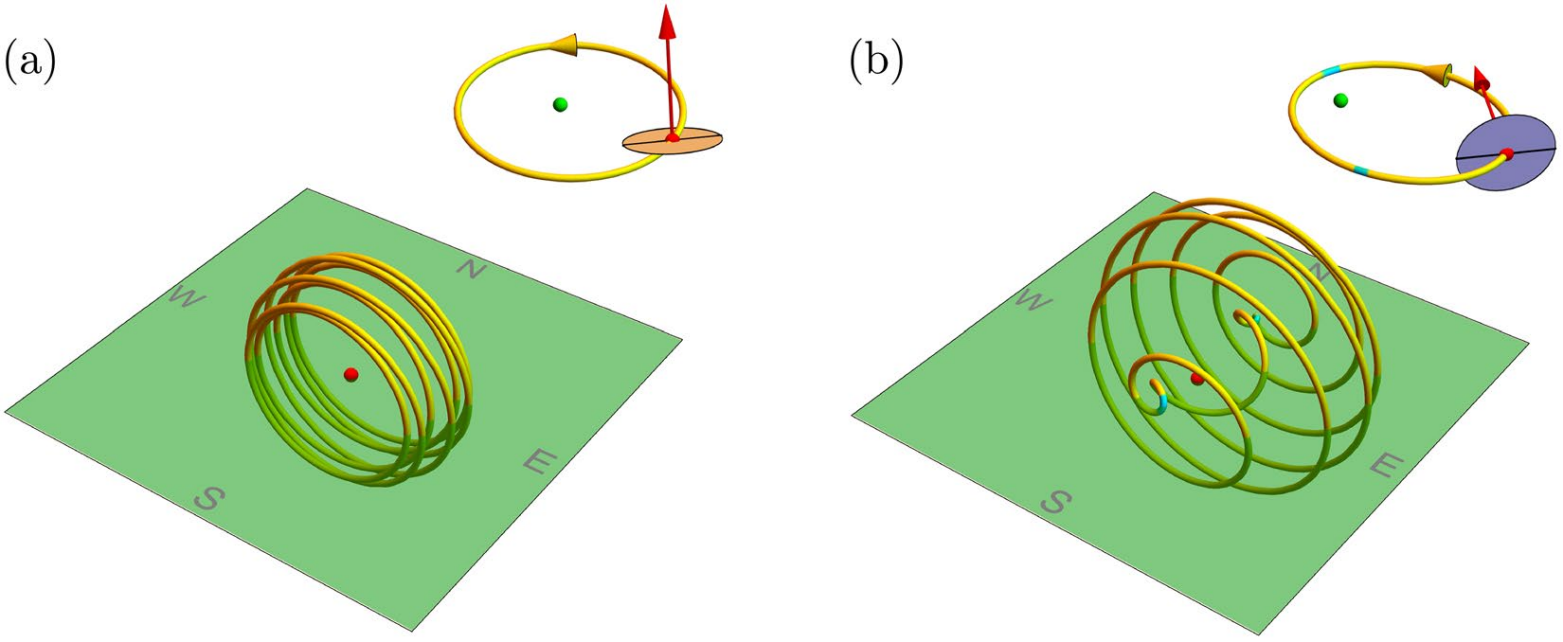
Ball plots. Dots (red) represent observers on their local horizons (green). (**a**) Earth-like planet with $\{\rho , e, \epsilon , \Omega \} = \{8, 0.1, 0.4, 3.8\}$, apparent solar motion (left) and orbit (right), with no reversals. (**b**) Exoplanet with $\{8, 0.6, 1.5, 3.8\}$, with apparent solar motion (left) and orbit (right), including reversals (cyan) not at periapsis.

## With tilt

More generally, consider a planet whose spin is tilted by an obliquity angle $\epsilon$ with respect to the orbital angular momentum vector $\vec L \propto {\hat{z}}$ and whose equatorial and orbital planes intersect at a nodal angle $\Omega$ with respect to the orbital eccentricity vector (or long axis of the elliptical orbit) $\vec \epsilon \propto {\hat{x}}$. At time *t*, an equatorial observer has a local north-up-east coordinate system

16a $$\begin{aligned} {\hat{N}}&= {\mathbf {R}}_{(\Omega -\pi /2){\hat{z}}} {\mathbf {R}}_{\epsilon {\hat{y}}} {\hat{z}}, \end{aligned}$$

16b $$\begin{aligned} {\hat{U}}&= {\mathbf {R}}_{(\Omega -\pi /2){\hat{z}}} {\mathbf {R}}_{\epsilon {\hat{y}}} {\mathbf {R}}_{\omega t {\hat{z}}} {\hat{x}}, \end{aligned}$$

16c $$\begin{aligned} {\hat{E}}&= {\hat{N}} \times {\hat{U}}, \end{aligned}$$

where ${\mathbf {R}}_{\phi {\hat{u}}}$ is a rotation matrix through an angle $\phi$ about a direction ${\hat{u}}$. More explicitly, the local unit vectors are

17a $$\begin{aligned} {\hat{N}}&= \{ \sin \epsilon \sin \Omega ,\ \sin \epsilon \sin \Omega ,\ \cos \epsilon \}, \end{aligned}$$

17b $$\begin{aligned} {\hat{U}}&= \{ \cos \Omega \sin \omega _s t + \sin \Omega \cos \epsilon \cos \omega _s t,\ \sin \Omega \sin \omega _s t - \cos \Omega \cos \epsilon \cos \omega _s t,\ -\sin \epsilon \cos \omega _s t \}, \end{aligned}$$

17c $$\begin{aligned} {\hat{E}}&= \{ \cos \Omega \cos \omega _s t - \sin \Omega \cos \epsilon \sin \omega _s t,\ \sin \Omega \cos \omega _s t + \cos \Omega \cos \epsilon \sin \omega _s t,\ \sin \epsilon \sin \omega _s t \}. \end{aligned}$$

We analyze apparent solar motion by using Mathematica to numerically integrate the Eq. (14) motion equations to find the planet’s location $\vec r$, compute the position of the sun

18 $$\begin{aligned} \vec r_s = - {\hat{U}} R - \vec r \end{aligned}$$

with respect to the observer, where $R \ll r$ is the planet’s radius, and the solar azimuthal angle

19 $$\begin{aligned} \alpha = \arctan \frac{\vec r_s \cdot {\hat{E}}}{\vec r_s \cdot {\hat{U}}}, \end{aligned}$$

where ${\dot{\alpha }} = d\alpha /dt < 0$ indicates a reversal.

**Figure 7:**
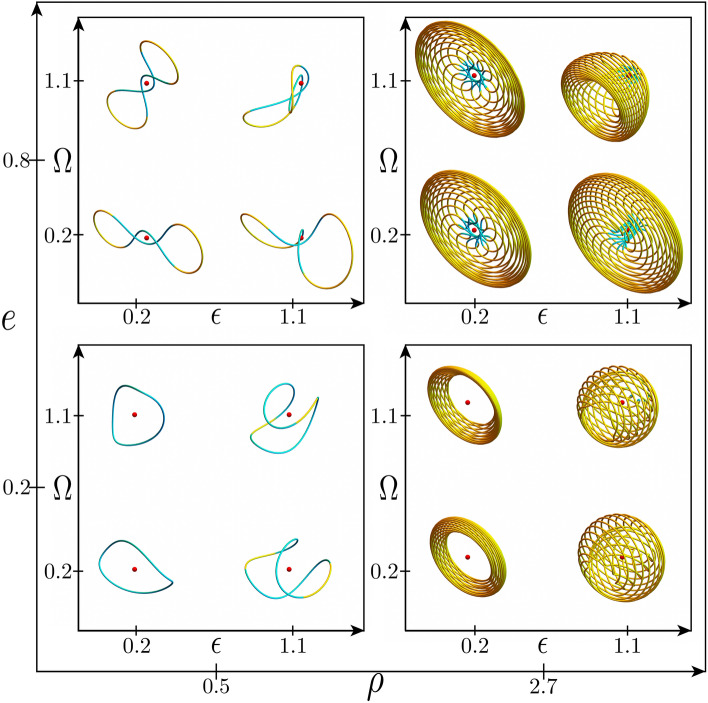
4D overview. Nested plot of plots of apparent solar motion (yellow with cyan reversals) relative to an equatorial observer (red dot) for representative eccentricity *e* versus spin–orbit ratio $\rho$ (outer) and nodal angle $\Omega$ versus obliquity $\epsilon$ (inners).

The 3D Fig. 5a plot shows the region of reversals as a function of orbital eccentricity $\epsilon$, spin–orbit ratio $\rho = \omega _s/\omega _o$, and obliquity $\epsilon$, for nodal angle $\Omega = 0$. For planets or satellites that maintain significant obliquities, dynamical evolution and forced nodal precession can lead to capture into long-term or secular spin–orbit resonance known as Cassini states^11^. Such planets maintain obliquity despite tidal dissipation, and the equilibrium rotation rate is a function of eccentricity and obliquity^12^, as indicated by the Fig. 5a checkered surface, all of which undergo reversals. The 2D Fig. 5b plot shows that small obliquities do not significantly change the reversals, where the dashed line represents Earth’s $\epsilon = 23.44^\circ \sim 0.4$ obliquity, although large obliquities expand the range. Dot-dashed curve indicates Cassini states. Similarly, small changes in the nodal angle $\Omega$ have a small effect.

We visualize the apparent solar motion by the Fig. 6 spatial “ball” plots. A dot (red) represents the observer on the local horizon (green). A tube represents the apparent solar motion, both forward (yellow) and backward (cyan). Inset (top-right) represents orbital motion in the solar system’s reference frame, where spin (red arrow) is tilted and dot (green) is the sun. Figure 6a illustrates an Earth-like planet whose sun apparently rises in the east and sets in the west with altitude varying with season and with no reversals; parameters are $\{\rho , e, \epsilon , \Omega \} = \{8, 0.1, 0.4, 3.8\}$. Figure 6b illustrates a non-Earth-like planet in a more eccentric orbit with a more oblique tilt whose sun rises in the east and sets in the west but reverses twice a year and *not* near periapsis; parameters are $\{8, 0.6, 1.5, 3.8\}$. The Fig. 7 nested plots of $\{\epsilon ,\Omega \}$ at each $\{\rho , e\}$ summarize the scope of solar motion. Again, moderate tilt is secondary to orbital eccentricity and spin–orbit ratio in determining the passage of the sun in the sky, including its reversals.

**Figure 8:**
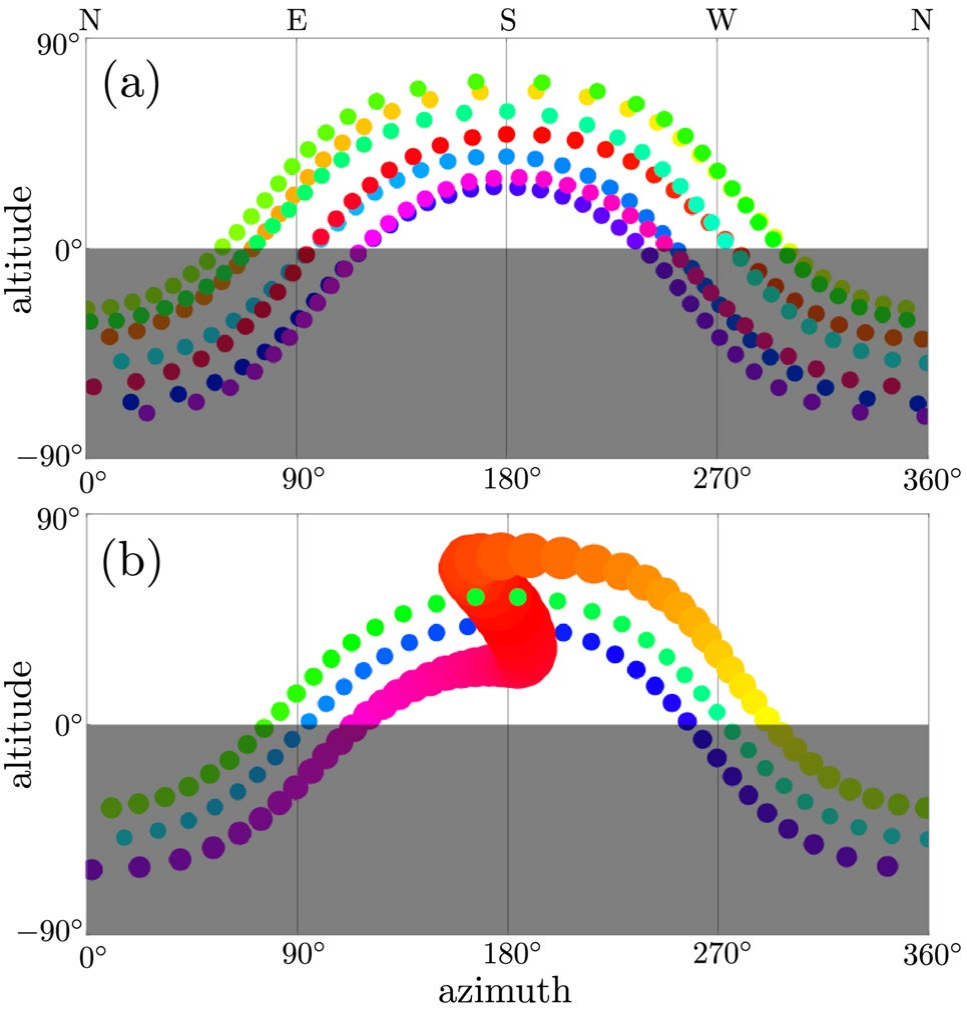
Solar strobe plots. (**a**) Earth-like planet whose sun appears to rise in the east and set in the west every day; rainbow hues code time. (**b**) Mercury-like exoplanet whose sun appears to reverse its motion once a year near periapsis where it appears largest; parameters are eccentricity $e = 0.675$, spin–orbit ratio $\rho = 1.5$, obliquity $\epsilon = 23.5^\circ$, nodal longitude $\Omega = 0$, observer co-latitude $\theta _{\mathscr {O}} = 60^\circ$, and observer longitude $\phi _{\mathscr {O}} = 180^\circ$.

Nonequatorial observers can also experience solar reversals. Figure 8a simulates the view from an Earth-like planet whose sun appears to rise in the east and set in the west every day for a year; rainbow hues code time and the sun is drawn at equal time intervals. Figure 8b simulates a Mercury-like exoplanet, nicknamed planet “Z”, whose sun appears to reverse its motion just once a year at noon and at periapsis where the sun appears largest; parameters are spin–orbit ratio $\rho = 1.5$, eccentricity $e = 0.675$, obliquity $\epsilon = 0.4$, nodal longitude $\Omega = 0$, observer co-latitude $\theta _{\mathscr {O}} = 60^\circ$ and longitude $\phi _{\mathscr {O}} = 180^\circ$.

## Apoday

For small obliquity, which may be common and includes the exoplanet $\beta$ Pictoris b^13^, and where the planar nature of the geometry even admits convenient analysis using complex numbers^14^, the without-tilt results suffice and reversals occur only near periapsis, as in the Fig. 8b strobe plot. A sun might even appear to rise in the east and set in the west, and then rise in the west for a “reverse” or “anti” day before setting in the east and repeating.

For planets in eccentric orbits with small obliquity and constant spin angular speed between their extreme orbital angular speeds, many “normal” days, when the planet orbits slowly near its apopapsis, will be punctuated once a year by a reversal day, when the planet orbits quickly near its periapsis, and the sun appears to crisscross the local meridian three times, as in Fig. 8b. For such cases, the notion of a solar day as the time between successive noons might need to be revised or discarded. We suggest defining the (variable) *apoday* to be the time between successive solar meridian crossings in the dominant, apoapsical direction. We expect solar reversals to have enormous cultural implications for any intelligent life that witnesses them.

## Discussion

The motion of alien suns in exoplanet skies, long the subject of science fiction^15^, is now becoming a focus of science fact. For example, observations^16^ by the recently launched James Webb Space Telescope of the periapsis heating of the high-eccentricity exoplanet HD 80606 b should elucidate its potential for both pseudosynchronization and solar reversals.

We do not yet know what fraction of exoplanets experience solar reversals and for how long. In our solar system, only Mercury experiences reversals, and just barely. The striking spin–orbit resonance and large eccentricity that make this possible may reflect a history (and future) of chaotic dynamics^5,17^. Also in our solar system, observers on Hyperion see Saturn and the sun rise and set chaotically^18^, while observers on Nix and Hydra see Pluto and the sun rise and set chaotically^19^. Similarly, some exoplanets may experience chaotic sunsets, at least for some of their lifetimes (as eccentricities, spins, and tilts can wander and evolve).

Exoplanets may be stable in multiple star systems^20^, where multiple reversals may be common. The recently discovered sextuply-eclipsing sextuple star system^21^ suggests dramatic possibilities. Like the growing interest in planetary habitability^22^ and alien skies^23^, knowledge of alien sunsets, sunrises, and solar motion enlarges our conception of possible worlds. Study of potential and other solar systems provides context for study of our own.

## Data Availability

A Mathematica notebook that generates most of the results in this article is available from the corresponding author on reasonable request.
